# Efficacy of paracetamol for the treatment of patent ductus arteriosus in preterm neonates

**DOI:** 10.1186/1824-7288-40-21

**Published:** 2014-02-20

**Authors:** Gianluca Terrin, Francesca Conte, Antonella Scipione, Erica Bacchio, Maria Giulia Conti, Rosalia Ferro, Flavia Ventriglia, Mario De Curtis

**Affiliations:** 1 Department of Gynecology-Obstetrics and Perinatal Medicine, University “La Sapienza”, Viale del Policlinico 155, 00161 Rome, Italy; 2 Department of Pediatrics, University “La Sapienza”, Rome, Italy

**Keywords:** Patent ductus arteriosus, Paracetamol, COX-inhibitors, Ibuprofen, Indomethacin

## Abstract

Inhibitors of the cyclo-oxygenase component of prostaglandin-H_2_ synthetase, namely indomethacin and ibuprofen, are commonly used in the treatment of hemodynamically significant patent ductus arteriosus. These drugs are associated with serious adverse events, including gastrointestinal perforation, renal failure and bleeding. The role of paracetamol, an inhibitor of the peroxidase component of prostaglandin-H_2_ synthetase, has been proposed for the treatment of patent ductus arteriosus. We report a series of 8 neonates (birth weight: 724 ± 173 g; gestational age: 26 ± 2 weeks) treated with paracetamol for a hemodynamically significant patent ductus arteriosus, because of contraindications to ibuprofen or indomethacin. Successful closure was achieved in 6 out of 8 babies (75%). Median ductal diameter was significantly reduced after treatment (from 1.2 mm, range 1.0-2.5 mm to 0.6 mm, range 0.0-2.5 mm, p = 0.038). No adverse or side effects were observed during treatment. On the basis of these results, paracetamol could be considered a promising and safe therapy for the treatment of patent ductus arteriosus in neonates.

## Background

A persistently patent ductus arteriosus (PDA) has significant clinical consequences in preterm neonates during the recovery period from respiratory distress syndrome [1]. Ductal patency is regulated by the circulating prostaglandins (PGs) produced by an enzyme system, namely prostaglandin-H_2_ synthetase (PGHS), which is composed of two active sites: cyclo-oxygenase (COX) and peroxidase [2,3]. Indomethacin and ibuprofen are COX-inhibitor drugs commonly used for the treatment of hemodynamically significant (hs)-PDA. Despite the about 70% success rate, COX-inhibitors are frequently contraindicated in early life and their use has been associated with serious adverse events, such as gastrointestinal perforation, renal failure and bleeding [4-11]. Paracetamol, an inhibitor of the peroxidase component of PGHS, is commonly used in pediatric age, and has been recently proposed for the treatment of PDA [12-22]. We aimed to evaluate the efficacy of paracetamol in the early treatment of PDA in preterm neonates presenting contraindication to COX-inhibitors.

## Case presentation

We report a case series of neonates with hs-PDA treated with paracetamol because of contraindication to ibuprofen or indomethacin, who were observed at the Neonatal Intensive Care Unit of the University of Rome “Sapienza”, from January 2012 to October 2013. During this study period, based on our policy, neonates with gestational age (GA) at birth < 32 weeks were evaluated daily to detect the presence of hs-PDA. Neonates with hs-PDA were treated with paracetamol in the presence of contraindications to ibuprofen or indomethacin (i.e. urine output < 1 ml/kg/h, intraventricular hemorrhage, platelet count < 60,000/mm^3^, hyperbilirubinemia requiring exchange transfusion, signs of feeding intolerance or gastrointestinal bleeding). The condition of hs-PDA was defined by the presence of at least one of the following criteria: internal ductal diameter ≥ 1.5 mm, left-atrium-to-aortic-root ratio > 1.6, unrestrictive pulsatile transductal flow, reverse or absent diastolic flow in the descending aorta [23]. Paracetamol was given at doses ranging from 7.5 to 15 mg/kg every 4–6 hours, with a maximum daily dose of 60 mg/Kg. We collected, in a specific reporting form, data regarding GA, sex, mode of delivery, birth weight (BW), Apgar score at 5 minutes, weight at enrollment, age at enrollment, contraindication to COX-inhibitor use and echocardiographic characteristics of PDA before and after therapy, obtained from the clinical charts and nursing records. Efficacy was defined by the rate of patients in whom we observed ductal closure (defined by the absence of shunt or diameter < 0.5 mm without any other hemodynamic implications) at echocardiographic examination performed daily during the study period. To monitor safety of paracetamol treatment, we collected data regarding serum concentration of liver enzymes, total and direct bilirubin, creatinine and urea nitrogen. Occurrence of common morbidities of prematurity (i.e. bronchopulmonary dysplasia, intraventricular hemorrhage, necrotizing enterocolitis), fatal events and side effects were also collected. The study was approved by the ethics committee of University of Rome La Sapienza. The χ^2^ test and Fisher’s exact test were used for categorical variables. The level of significance for all statistical tests was 2-sided, *p* < 0.05. Statistical analysis was performed with SPSS Version 16.0 for Windows (SPSS Inc., Chicago, IL).

During the study period we observed 8 neonates treated with paracetamol for an hs-PDA. Successful closure was obtained in 6 out of 8 neonates. Median ductal diameter was significantly reduced after treatment (from 1.2 mm, range 1.0-2.5 mm to 0.6 mm, range 0.0-2.5 mm, p = 0.038). Baseline clinical findings, contraindications to COX-inhibitors and echocardiographic characteristics of our study population are reported in Table 1.

**Table 1:** Main demographic, clinical and echocardiographic findings of preterm neonates before treatment with paracetamol

**Characteristics at baseline**	
**Demographic and clinical features**	
N.	8
Body weight at birth, g	724 ± 173
Gestational age at birth, weeks	26 ± 2
Caesarian section, n (%)	6 (75.0)
Male, n (%)	6 (75.0)
Apgar at 5 minutes	5 ± 1
Resuscitation at birth, n (%)	8 (100)
Body weight at enrollment, g	743.7 ± 167.6
Age at enrollment, h	66 ± 28
**Contraindication to COX-inhibitors**	
Oliguria, n (%)	4 (50.0)
Thrombocytopenia, n (%)	2 (25.0)
Intraventricular hemorrhage, n (%)	1 (12.5)
Blood in gastric aspirates, n (%)	1 (12.5)
Hyperbilirubinemia requiring phototherapy, n (%)	4 (50.0)
Hyperbilirubinemia requiring exchange transfusion, n (%)	0 (0.0)
**Echocardiographic findings**	
Diameter of the ductus ≥ 1.5 mm, n (%)	3 (37.5)
Left-atrium-to-aortic-root ratio > 1.6, n (%)	6 (75.0)
Unrestrictive pulsatile transductal flow, n (%)	1 (12.5)
Reverse flow in the descending aorta, n (%)	2 (25.0)

Notes. Data are expressed as mean ± standard deviation, when not specified.

The two patients in whom paracetamol failed to close the ductus, intravenous ibuprofen (at a dose of 10 mg/kg followed by 5 mg/kg after 24 and 48 hours) was given considering that previous contraindications were no longer present. Surgical ligation was finally needed for both patients after ibuprofen failure. None of our patients experienced re-opening of the ductus after closure. No adverse or side effects were observed during treatment (Table 2). One of the 2 patients who failed treatment with paracetamol developed bronchopulmonary dysplasia.

**Table 2:** Serum level of liver enzymes and bilirubin before and after treatment with paracetamol

	**At baseline**	**At end of therapy**	***p***
**Safety**			
Alanine aminotransferase, U/L	56 (8-78)	55 (4-78)	0.878
Aspartate aminotransferase, U/L	54 (26-80)	31 (8-62)	0.015
Bilirubinemia, mg/dl	7.6 (3.4-12.8)	5.0 (2.7-7.5)	0.050

Notes. Data are expressed as median (range: upper - lower values).

## Conclusions

Our case series demonstrates the efficacy of paracetamol in hs-PDA in preterm neonates with contraindication to COX inhibitors. Nine case series involving a total number of 67 neonates treated with paracetamol for PDA, have been previously reported [12-20]. The larger series evaluating efficacy of paracetamol in a similar setting, included a maximum number of 10 patients [13,16,20]. Six studies verified the efficacy of paracetamol in very low birth weight neonates [12,13,16,17,19,20]; four of them reported a rate of ductal closure of 71-100% [12,13,16,17], while 2 series observed a very low efficacy rate (i.e. 0-20%) [19,20]. In particular, Alan S. et al. reported the failure of paracetamol in 3 neonates treated with this drug after one or more unsuccessful courses of ibuprofen [19]. In the case series reported by Roofthooft et al., paracetamol was ineffective in 8 out of 10 neonates [20]; however, these patients had a lower GA and BW [20] and started paracetamol treatment later [19,20] than the patients in our case series. All these data suggest that lower GA and BW associated with previous courses of ibuprofen or later ductal treatment may negatively influence the response to paracetamol [24-26]. Accordingly, we observed a failure of paracetamol treatment in 2 subjects with very low GA. On the other hand, the high rate of ductal closure observed in our population could be associated with the earlier start of paracetamol treatment compared with the vast majority of the previous reports.

In five previous studies, paracetamol was administered by enteral route [12,14-16,18] and in 4 by intravenous route [13,17,19,20]. All of our patients were given paracetamol intravenously because they presented signs of feeding intolerance (i.e., gastric residuals, vomiting, abdominal distension). Even if parenteral route is associated with higher peak and less variability of plasma concentration [27], apparently a similar rate of ductal closure was observed also in reports using oral paracetamol. High doses (60 mg/kg/day) of paracetamol were used in 7 out of 9 previous studies [12-15,18-20], while 2 studies reported the administration of lower doses of paracetamol (30–45 mg/kg/day), apparently without any reduction in efficacy [16,17]. However no study comparing different dosing regimens is available at the moment.

The mechanism of action of paracetamol remains largely uninvestigated. Paracetamol, by inhibiting PG production [3], firstly induces smooth muscle constriction and narrowing of the lumen, similarly to COX-inhibitors. In the subsequent phases of ductal closure, platelet aggregation plays a crucial role in the formation of a thrombus that occludes ductal lumen [28,29]. A lower anti-platelet activity of paracetamol compared to that of COX-inhibitors may positively influence this phase of the closure process [3]. In any case, future studies should be performed to shed light on the mode of action of paracetamol.

Although interesting, the results of our study suffer from some limitations. Similarly to previous case series, ours is based on a limited number of neonates. Recently, two randomized clinical trials have demonstrated similar efficacy of oral paracetamol compared with oral ibuprofen [21,22]. Although interesting, results of these trials do not clarify many aspects related to paracetamol use in neonatal age (i.e., optimal dosage, time to start therapy and route of administration). In addition these trials present significant limitations such as inadequate sample power calculation and absence of per intention-to-treat analysis. Pharmacokinetic studies were not performed in our population to avoid adjunctive procedures and blood collections in preterm infants at high risk of iatrogenic anemia [30,31]. Nevertheless previous studies have demonstrated that the doses used in our study are safe and associated with optimal levels of serum paracetamol concentrations [13].

Our results encourage further well designed studies aimed at verifying the efficacy of paracetamol also as first choice agent in the treatment of PDA.

## Consent

Written informed consent was obtained from the parents of the patients for the publication of this report.

## Abbreviations

BW: Birth weight; COX: Cyclo-oxygenase; GA: Gestational age; Hs-PDA: Hemodynamically significant-patent ductus arteriosus; PDA: Patent ductus arteriosus; PGHS: Prostaglandin-H_2_ synthetase; PGs: Prostaglandins.

## Competing interests

All authors declare that they have no potential, perceived, or real conflict of interest to disclose. No honorarium, grant, or other form of payment was given to anyone to produce this manuscript.

## Authors’ contributions

GT, MDC and FV designed the study; GT and FC performed data monitoring and statistical analysis; AS, EB, FC, MGC and RF cared for the patients, collected data and wrote the first draft of the manuscript; FV performed echocardiographic evaluations and all the authors revised and approved the final version of the paper.
